# Design, optimization, and *in vitro* antimicrobial evaluation of a clove leaf oil nanoemulsion against bovine mastitis-associated pathogens

**DOI:** 10.14202/vetworld.2026.1257-1272

**Published:** 2026-03-23

**Authors:** Afduha Nurus Syamsi, Suyadi Suyadi, Lilik Eka Radiati, Tri Eko Susilorini

**Affiliations:** 1 Doctoral Student in Animal Science, Faculty of Animal Science, Universitas Brawijaya, Malang 65145, Indonesia; 2 Department of Animal Science, Faculty of Animal Science, Universitas Jenderal Soedirman, Purwokerto 53122, Indonesia; 3 Department of Animal Science, Faculty of Animal Science, Universitas Brawijaya, Malang 65145, Indonesia; 4 Department of Animal Product Technology, Faculty of Animal Science, Universitas Brawijaya, Malang 65415, Indonesia

**Keywords:** antimicrobial activity, bovine mastitis, clove leaf oil, nanoemulsion, self-nanoemulsifying drug delivery system, *Staphylococcus aureus*, teat disinfectant, *in vitro*

## Abstract

**Background and Aim:**

Bovine mastitis is one of the most economically significant diseases affecting dairy cattle, leading to substantial losses due to decreased milk yield, higher treatment costs, and lower milk quality. Iodine-based teat disinfectants are commonly used to prevent mastitis but have several limitations, such as milk residue contamination, potential skin irritation, environmental persistence, and concerns about antimicrobial resistance. Plant-derived antimicrobials have thus gained interest as potential alternatives. Clove leaf oil from Syzygium aromaticum (L.) Merr. & L.M. Perry contains eugenol and other bioactive compounds with strong antimicrobial activity. However, its use is limited by poor water solubility and physicochemical instability. This study aimed to develop and optimize a clove leaf oil nanoemulsion (CLON) using a self-nanoemulsifying drug delivery system (SNEDDS) and to evaluate its physicochemical properties and in vitro antimicrobial activity against pathogens associated with bovine mastitis.

**Materials and Methods:**

This study followed a three-phase sequential workflow. Phase I involved screening and optimizing the formulation of CLON using SNEDDS technology. Nine ratios of clove leaf oil, Polysorbate 80 (Tween 80), and Polyethylene glycol 400 (PEG 400) were initially tested, followed by optimization with an I-optimal mixture design. Phase II included physicochemical characterization and assessing the thermodynamic stability of the optimized nanoemulsion, such as measurements of transmittance, pH, emulsification time, droplet size, polydispersity index, zeta potential, and transmission electron microscopy morphology. Phase III examined the in vitro antimicrobial activity of CLON against Staphylococcus aureus, Escherichia coli, and Candida albicans through agar diffusion assays, determination of minimum inhibitory concentration (MIC), minimum bactericidal concentration (MBC), and killing-time kinetics. Inhibition zone data were analyzed using analysis of variance, followed by Duncan’s multiple range test.

**Results:**

The optimized CLON formulation contained 14.29% clove leaf oil, 68.56% Tween 80, and 17.15% PEG 400. The nanoemulsion showed high optical clarity (93.36 ± 0.25%), physiological pH (7.13 ± 0.01), rapid emulsification (35.46 ± 0.08 s), nanoscale droplet size (25.19 ± 2.31 nm), low polydispersity index (0.30 ± 0.04), and strong negative zeta potential (−45.07 ± 8.15 mV), indicating excellent stability. CLON demonstrated significant concentration-dependent antimicrobial activity (p < 0.01) against all tested microorganisms. The MIC and MBC values were 12.5% and 50% (v/v), respectively, indicating bactericidal activity. In the killing-time assay, 25% CLON achieved >3 log_10_ colony-forming units/mL reduction within 60 min.

**Conclusion:**

The optimized CLON displayed favorable physicochemical properties, strong thermodynamic stability, and broad-spectrum in vitro antimicrobial activity against key bovine mastitis pathogens. These results indicate that CLON has potential as a plant-based alternative teat disinfectant to prevent mastitis. However, further in vivo studies and field trials are needed to verify its safety and practical use in dairy production systems.

## INTRODUCTION

Mastitis is an infection of the mammary gland in dairy cattle, primarily caused by *Staphylococcus aureus*, *Escherichia coli*, *Candida albicans*, and other minor microorganisms. It reduces milk production (28.4%–53.5% per lactation), raises treatment costs (5%–25% annually), and results in significant yearly economic losses of at least USD 662 per cow. Iodine-based pre-milking teat disinfection is one of the most effective control strategies, decreasing mastitis prevalence by about 60%. However, concerns remain about milk residue contamination (21 μg/L), the potential development of antimicrobial resistance, skin irritation on teat surfaces, and environmental persistence [1–3]. These issues emphasize the need for alternative plant-based agents with broad-spectrum antimicrobial properties to replace or supplement conventional synthetic disinfectants.

Clove leaf oil (CLO) is a promising natural antimicrobial agent for teat disinfection. Unlike clove bud oil, CLO is derived from fallen leaves, an agricultural byproduct, making it a more sustainable and cost-effective raw material for large-scale farm use. Its high eugenol content (74.28%–94.41%), similar to that of clove bud oil, along with other minor phytochemicals, enhances its antimicrobial activity through rapid membrane disruption and multi-target cellular interference. These features are advantageous for teat disinfectant formulations that require quick antimicrobial action. However, CLO has some limitations, including poor water solubility, physicochemical instability, and low bioavailability, which hinder its direct therapeutic or disinfectant use [4]. Self-nanoemulsifying drug delivery systems (SNEDDS) offer several benefits over traditional high-energy emulsification methods, such as spontaneous emulsification at room temperature (27°C ± 2°C), easier processing without specialized equip-ment, and better scalability. These qualities can help translate the technology to practical farm applications [5].

Previous SNEDDS-based studies involving cardamom and lemongrass essential oils reported excellent nanoemulsion characteristics, including droplet sizes of 13.17–13.97 nm, low polydispersity index values (0.06–0.17), and good physical stability [6, 7]. Similarly, clove oil nanoemulsions with droplet sizes of 59–134 nm have shown strong inhibitory effects against Gram-positive (*S. aureus*) and Gram-negative (*E. coli*) bacteria [8], whereas formulations with droplet sizes of 32.67–225.8 nm exhibited antifungal activity against *C. albicans* [9]. However, most previous studies mainly focused on food preservation applications using high-energy homogenization techniques. These studies generally reported only inhibition zone results without evaluating time–kill kinetics relevant to disinfectant performance standards, primarily used clove bud oil rather than the more sustainable leaf oil, and did not consider physiological compatibility with bovine teat skin pH (7.00–7.18) as a formulation criterion. These limitations emphasize the need for a specifically designed CLO nanoemulsion (CLON) optimized for mastitis teat disinfection applications.

The novelty of this study is in using CLO as a sustainable replacement for clove bud oil, optimizing formulations systematically with an I-optimal mixture design to achieve ultra-small droplet sizes without high-pressure homogenization, ensuring physiological pH compatibility with bovine teat skin, and conducting a thorough antimicrobial evaluation including time–kill kinetics to meet disinfectant performance standards.

Therefore, this study developed a SNEDDS-optimized CLON specifically aimed at bovine mastitis teat disinfection. Unlike conventional antimicrobial nanoemulsions designed for therapeutic drug delivery, this CLON formulation was created for disinfectant use, emphasizing rapid antimicrobial action, neutral pH compatibility with bovine teat skin, and formulation stability under farm conditions. As a preclinical formulation and screening study, the originality lies in the formulation design approach, systematic optimization, and proof-of-concept demonstration rather than in producing a final product. Consequently, this study aimed to develop an optimized CLON using SNEDDS and to evaluate its physicochemical properties, stability, and *in vitro* antimicrobial effectiveness against standard reference strains representing pathogens associated with bovine mastitis.

## MATERIALS AND METHODS

### Ethical approval

This *in vitro* study used commercially available American Type Culture Collection (ATCC) reference strains and did not involve live animals, clinical samples, or human subjects. Therefore, ethical approval was not needed.

### Study period and location

The study was conducted from January to March 2025 at the Dairy Production Laboratory, Universitas Jenderal Soedirman, Purwokerto, Central Java, Indonesia.

### Study design

This study followed a three-phase sequential workflow. First, nine CLO:surfactant:co-surfactant ratios were tested for formulation screening to find candidates that meet the clarity criterion (transmittance >90%), followed by optimization using an I-optimal mixture design. Second, the optimized formulation underwent physicochemical characterization and thermodynamic stability testing. Third, the *in vitro* antimicrobial activity of the stable CLON against bovine mastitis-associated pathogens was assessed.

### Materials

The main materials included CLO (from a local commercial distiller, Cilacap, Indonesia), Tween 80 (≥95%, Iberchem, Jakarta, Indonesia), and Polyethylene glycol (PEG) 400 (≥95%, Green Pharma, Selangor, Malaysia). Mueller–Hinton Agar and broth (MHA/MHB), Sabouraud Dextrose Agar (SDA), and RPMI-1640 were sourced from HiMedia (Mumbai, India). Cultures of *Staphylococcus aureus* ATCC 6538P, *Escherichia coli* ATCC 25922, and *Candida albicans* ATCC 14053 (PT. Agritama Sinergi Inovasi, Bandung, Indonesia) were stored at 4°C with monthly subculturing and revived on MHA (37°C, 24 h) for bacteria and SDA (30°C, 48 h) for yeast. Other materials included deionized water (pH 6.8–7.0) and physiological saline (0.9% NaCl).

The instruments used included an ultrasonic bath (35 W, 0.8 L capacity), Digital Hotplate Magnetic Stirrer-Homogenizer H3770-HS (Benchmark Scientific, Sayreville, NJ, USA), Multiskan Sky UV–Vis microplate spectrophotometer (Thermo Fisher Scientific, Waltham, MA, USA), Shimadzu GC–MS-QP2020NX (Kyoto, Japan), digital pH meter (Hanna HI991001, Woonsocket, RI, USA), particle size analyzer (Microtrac Nanotrac Wave II, Montgomeryville, PA, USA), JEOL JEM-1400 Transmission Electron Microscope (Akishima, Tokyo, Japan), and Hettich ROTOFIX 32 A centrifuge (Tuttlingen, Germany).

### CLO extraction

CLO was extracted from fallen leaves of *Syzygium aromaticum* (L.) Merr. & L. M. Perry, botanically authenticated in January 2026 at the Herbarium of the Biology Faculty, Universitas Jenderal Soedirman, Purwokerto, Indonesia (voucher No. PUNS-Syamsi-001). The collected leaves were washed and sun-dried for 2–3 days, then steam-distilled in a mild steel vessel (water capacity ~500 L, batch size ~200 kg) for 6–8 h at approximately 100°C, producing 1.5%–3.0% oil (dry weight basis). The crude oil was treated with 5% citric acid to chelate iron contaminants, followed by overnight treatment with 10% anhydrous Na_2_SO_4_ to remove residual water, and then decanted to obtain a pale yellow oil [10].

The essential oil components were analyzed by GC–MS using an HP-5MS capillary column with helium as the carrier gas (1.00 mL/min). Split injection (10:1) was performed at 250°C, and the oven temperature was programmed from 50°C to 250°C at 5°C/min. Compounds were identified in scan mode (m/z 35–600) using the NIST database (Table 1).

**Table 1:** Gas chromatography–mass spectrometry identification of clove leaf essential oil.

No.	Area (%)	RT (minutes)	Components	No.	Area (%)	RT (minutes)	Components
1	72.90	20.675	Eugenol	18	0.16	27.485	Caryophylladienol
2	12.00	21.860–22.218	β-Caryophyllene	19	0.13	25.954	Caryophyllenol
3	3.84	23.068	Eugenyl acetate	20	0.12	22.412	Isocaryophyllene
4	2.12	25.626–26.244	Caryophyllene oxide	21	0.12	23.526	α-Amorphene
5	1.88	21.044	α-Copaene	22	0.11	15.537	Terpinen-4-ol
6	0.90	24.740	δ-Selinene	23	0.10	10.815	m-Cymene
7	0.88	25.493–28.252	14-Hydroxycaryophyllene	24	0.10	13.175	Linalool
8	0.72	21.259	Methyl cinnamate	25	0.09	9.660	Sulcatone
9	0.70	7.984	α-Pinene	26	0.08	10.342	δ-3-Carene
10	0.56	20.298	α-Cubebene	27	0.08	24.976	α-Muurolene
11	0.45	9.306	β-Pinene	28	0.06	23.242	δ-Cadinene
12	0.28	26.857	Humulene epoxide II	29	0.05	10.547	α-Terpinene
13	0.21	10.933	Limonene	30	0.05	15.951	α-Terpineol
14	0.21	16.045	Methyl salicylate	31	0.04	9.747	Myrcene
15	0.20	21.758	Methyl eugenol	32	0.04	13.621	DMNT
16	0.20	24.262	α-Farnesene	33	0.04	21.444	β-Elemene
17	0.19	17.883	Chavicol	34	0.03	11.875	γ-Terpinene

RT = Retention time.

### Pre-formulation: SNEDDS ratio screening

CLON was developed using the SNEDDS method [6, 7]. Nine combinations of CLO:Tween 80:PEG 400 (ratios of 1:1:1 to 1:9:1, total volume 10 mL, v/v) were tested in triplicate. CLO was mixed with Tween 80 using a magnetic stirrer (500 rpm, 10 min, 27°C ± 2°C). This moderate stirring speed ensured thorough mixing while reducing air incorporation and thermal degradation of volatile compounds [11].

After adding PEG 400 and mixing for 10 min, the coarse emulsion was sonicated at 40 kHz (power density approximately 0.44 W/mL, based on 35 W output in an 80 mL working volume, at 40°C for 600 seconds). The 40 kHz frequency was chosen because it lies within the optimal range for creating nanoemulsions without causing excessive cavitation damage to essential oil components [12].

Formulations were visually assessed under ambient light [13], and transmittance was measured at 650 nm after dilution in deionized water (1:50 ratio; 200–300 μL per well) [6]. Formulations with clear appearance and transmittance >90% at the lowest surfactant ratio were selected [14].

### Optimization of CLON

The chosen formulation was optimized using an I-optimal mixture design with three components: X_1_ (CLO), X_2_ (Tween 80), and X_3_ (PEG 400), constrained to a total of 100%. The responses measured were transmittance (Y_1_, aiming for maximum), pH (Y_2_, aiming for 6.8–7.2), and emulsification time (Y_3_ aiming for less than 1 min). Data were analyzed with Design-Expert software version 13 [15, 16]. Model selection employed a linear sequential approach:

Transmittance was measured as described above. The pH was measured at 27°C ± 2°C using a calibrated digital pH meter (buffers pH 4.0 and 7.0) with a 5 mL sample. Emulsification time was determined by mixing 100 μL of CLON with 50 mL of physiological saline (0.9% NaCl, pH 7–7.4) under stirring at 100 rpm (37°C), and the time needed for the solution to become clear was recorded [17].

The optimized formulation was prepared as described in the pre-formulation step and stored at 27°C ± 2°C for characterization within 24 h.

### Characterization of CLON

Droplet size (DS), polydispersity index (PDI), and zeta potential (ZP) were measured with a particle size analyzer (PSA). Samples were diluted in deionized water (1:100 v/v) and gently homogenized (350 rpm, 30–60 s). Measurements were conducted in triplicate at 25°C using a scattering angle of 173° (backscatter), with deionized water as the dispersant (viscosity: 0.8872 cP; refractive index: 1.330).

Morphological characteristics were examined using transmission electron microscopy (TEM). Samples were diluted (1:1000) in deionized water, deposited onto Formvar-carbon-coated 300-mesh copper grids (Ted Pella, USA), and negatively stained with 2% phosphotungstic acid for 30 s before air-drying. Imaging was performed at an accelerating voltage of 100 kV with at least five randomly selected fields [16].

### Physical and thermodynamic stability tests

Physical stability was tested by centrifugation (1,006 × *g*, 30 min). Thermodynamic stability was evaluated through heating–cooling cycles (alternating between 4°C and 45°C, 48 h each, over three cycles) and freeze–thaw cycles (alternating between −4°C and 25°C, 48 h each, over three cycles). The temperature range of −4°C to 45°C was chosen to reflect typical farm storage conditions rather than extreme laboratory temperatures. Formulations had to pass all tests in sequence, and the absence of creaming, phase separation, or cracking confirmed stability [6]. This study centered on the development of nanoemulsion concentrates, while long-term storage stability and shelf-life modeling will be explored in future research.

### Antimicrobial activity

#### Agar diffusion assay

The inhibition zone was measured using the agar well-diffusion method following CLSI 2020 guidelines [18]. The experiment employed a completely randomized design with eight treatments: povidone iodine 1% (positive control), excipient control (Tween 80 + PEG 400), CLON 0%, CLON 20%, CLON 40%, CLON 60%, CLON 80%, and CLON 100%, each tested in triplicate.

Microbial suspensions were adjusted to McFarland 0.5 (~1.5 × 10^8^ CFU/mL for bacteria; ~1.5 × 10^6^ CFU/mL for yeast). The suspensions were spread onto MHA for bacteria or SDA for yeast using sterile swabs. Wells (6 mm diameter, 4 mm depth) were created using a sterile cork borer, and 100 μL of test solution was added to each well. Plates were kept at 27°C ± 2°C for 15–30 min to allow diffusion and then incubated at 37°C (bacteria) or 30°C (yeast) for 18–24 h. Inhibition zone diameters were measured using a digital caliper.

#### Minimum inhibitory concentration (MIC) and minimum bactericidal concentration (MBC) determination

MIC and MBC were determined using the broth microdilution method following CLSI 2020 guidelines [18]. CLON was serially diluted two-fold (100%, 50%, 25%, 12.5%, 6.25%, 3.125%, 1.56%, and 0.78% v/v) in MHB for bacteria or RPMI-1640 for yeast using 96-well microtiter plates. Povidone iodine 1% was similarly diluted as a positive control.

Standardized microbial suspensions (~5 × 10^5^ CFU/mL for bacteria; ~15 × 10^3^ CFU/mL for yeasts) were inoculated with 10 μL and incubated at 37°C for 18–24 h. MIC was determined spectrophotometrically as the lowest concentration that produced ≥90% growth inhibition (OD600). For MBC determination, 10 μL from wells without visible growth were subcultured onto MHA or SDA. MBC was defined as the concentration that caused ≥99.9% lethality. During povidone iodine testing, sodium thiosulfate (0.5%) was added to neutralize iodine carryover.

#### Killing-time assay

Killing-time assays were performed with *S. aureus*, *E. coli*, and *C. albicans* following CLSI M26-A guidelines [18]. Treatments included CLON 0% (negative control), 1× MIC, 2× MIC, 3× MIC, and povidone iodine 1% (positive control).

The starting inoculum (~1 × 10^6^ CFU/mL) was incubated with test solutions. At 5, 15, 30, and 60 min, samples were serially diluted (10^-4^) and plated onto MHA or SDA. Colonies were counted (30–300 range) and expressed as log_10_ CFU/mL.

The area under the killing curve (AUKC) was calculated using the trapezoidal rule:

*AUKC* = ∑[(*t*_*i*+1_-*t_i_*)×(*C_i_*+*C*_*i*+1_)/2]

Lower AUKC values indicated greater antimicrobial efficacy.

### Statistical analysis

Optimization data were analyzed using Design-Expert software version 13. Model adequacy was assessed through analysis of variance and lack-of-fit tests. Data normality was tested with the Shapiro–Wilk test. Data that were normally distributed are presented as mean ± standard deviation.

Antimicrobial assays were conducted in triplicate and repeated across three independent experiments. Inhibition zone data were analyzed using one-way analysis of variance followed by Duncan’s multiple range test. A paired t-test was employed to compare predicted and experimental values for model validation. MIC and MBC values were reported as the mode from triplicate determinations. Killing-time data were analyzed using regression assuming first-order kinetics.

## RESULTS

### Formulation and optimization of CLON

The initial screening of nine formulations with varying ratios of CLO, Tween 80, and PEG 400 revealed a range of visual appearances and transmittance values (Table 2). The first- to third-formulations appeared milky white to turbid and had low transmittance values (1.84%–54.53%), indicating unstable emulsions. In contrast, formulations four to nine appeared clear with high transmittance values (90.02%–91.69%), confirming the formation of transparent nanoemulsions. Among these, formulation four (ratio 1:4:1) was chosen for further optimization because it achieved the desired clarity with the lowest Tween 80 concentration.

**Table 2:** Initial screening of oil and Smix ratio in clove leaf oil nanoemulsion formulation.

Formula	CLO	Tween 80	PEG 400	Visual appearance	Average transmittance (%)
1	1	1	1	Milky white	1.84 ± 0.46
2	1	2	1	Milky	34.15 ± 2.98
3	1	3	1	Turbid	54.53 ± 0.30
4	1	4	1	Clear	90.02 ± 0.43
5	1	5	1	Clear	90.02 ± 0.32
6	1	6	1	Clear	91.34 ± 0.32
7	1	7	1	Clear	91.48 ± 0.12
8	1	8	1	Clear	91.69 ± 0.12
9	1	9	1	Clear	91.41 ± 0.21

The I-optimal mixture design was used to define the design space for formulation optimization. The lower, target, and upper limits of the independent variables were set based on component ratios of 1:3:1, 1:4:1, and 1:5:1. The lower boundary was established at 14.29%, and the upper boundary at 71.43%. A total of 17 experimental runs were performed (Table 3). Statistical analysis of the model and residual responses (Table 4) showed highly significant effects (p < 0.01) for all responses, including transmittance, pH, and emulsification time. The lack-of-fit test was not significant (p > 0.05), indicating good agreement between predicted and experimental values. Additionally, the predicted R² values closely matched the adjusted R² values for all responses (difference <0.2), confirming the model’s good predictability. The linear model equations revealed that Tween 80 (B) had the highest positive coefficient for transmittance, while CLO (A) had the greatest impact on emulsification time.

**Table 3:** Optimization of formulation design.

Sample	CLO (%)	Tween 80 (%)	PEG 400 (%)	Transmittance (%)	pH	Emulsification time (s)
1	20.00	63.56	16.44	55.87	7.12	37.46
2	18.74	62.69	18.57	55.34	7.10	36.99
3	17.11	65.14	17.75	77.75	7.11	36.47
4	14.29	68.56	17.15	93.54	7.14	35.40
5	14.89	70.82	14.29	93.18	7.12	35.52
6	20.00	65.42	14.58	74.50	7.07	37.35
7	17.11	65.14	17.75	83.83	7.12	36.43
8	17.11	65.14	17.75	83.95	7.09	35.43
9	19.32	60.68	20.00	66.48	7.11	37.29
10	16.30	68.89	14.80	89.74	7.12	36.09
11	20.00	63.56	16.44	71.29	7.08	37.36
12	14.77	65.23	20.00	92.68	7.16	35.73
13	14.29	68.56	17.15	92.40	7.12	35.39
14	18.30	67.41	14.29	75.12	7.02	36.77
15	19.32	60.68	20.00	67.69	7.11	37.30
16	16.70	63.30	20.00	81.78	7.12	36.30
17	16.22	67.22	16.55	90.23	7.11	36.10

CLO = Clove leaf oil, PEG = Polyethylene glycol.

**Table 4:** Analysis of the model and residual responses.

Response	F value	p-value	R²	Adjusted R²	Predicted R²
Transmittance (Model: Linear, Eq. T = 36.84A + 99.10B + 85.94C)	28.87	0.0000468	0.808	0.777	0.723
Residual (Lack-of-fit)	1.35	0.389	–	–	–
pH (Model: Linear, Eq. pH = 7.01A + 7.11B + 7.19C)	8.75	0.0034	0.556	0.492	0.313
Residual (Lack-of-fit)	2.42	0.172	–	–	–
Emulsification time (Model: Linear, Eq. ET = 39.36A + 35.28B + 35.54C)	61.20	0.0000009	0.897	0.883	0.879
Residual (Lack-of-fit)	0.20	0.98	–	–	–

A p-value < 0.05 was considered statistically significant.Eq. = Equation, T = Transmittance, ET = Emulsification time, A = Clove leaf oil, B = Tween 80, C = PEG 400, R² = Coefficient of determination.

**Figure 1:**
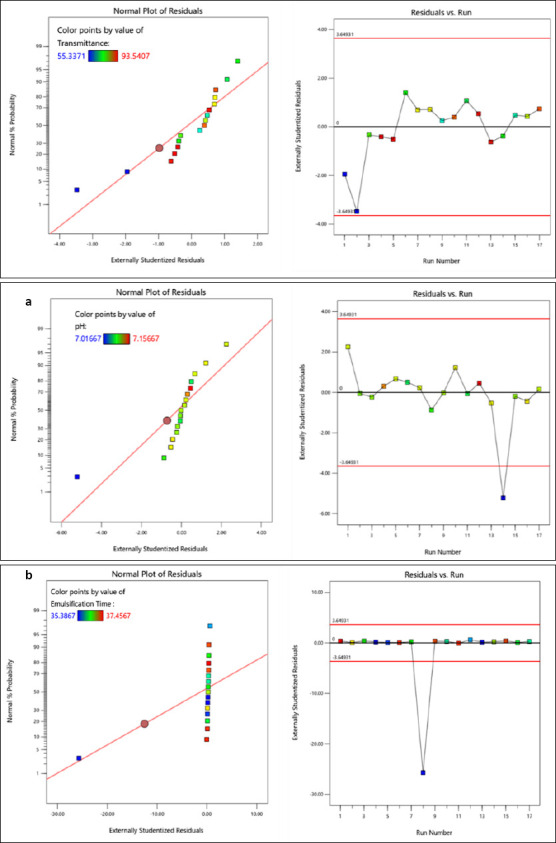
Residual parameters of (a) transmittance, (b) pH, and (c) emulsification time.

**Figure 2:**
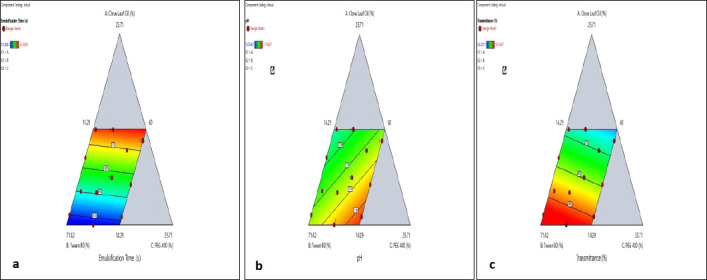
Pseudo-ternary contour plots showing the effects of clove leaf oil, Tween 80, and Polyethylene glycol 400 composition on (a) transmittance, (b) emulsification time, and (c) clove leaf oil nanoemulsion formulation pH.

Diagnostic plots for transmittance (Figure 1a), pH (Figure 1b), and emulsification time (Figure 1c) confirmed that the fitted linear models met key statistical assumptions. Table 5 shows the results of a single-sample t-test comparing predicted and experimental values to evaluate the predictive accuracy of the optimized formulations. The pH model (predicted: 7.11; actual: 7.13 ± 0.01; p = 0.236) and the emulsification time model (predicted: 35.91 s; actual: 35.46 ± 0.08 s; p = 0.072) demonstrated excellent agreement. Although the transmittance model showed a statistically significant difference (p = 0.021), the experimental value (93.36 ± 0.25%) was higher than the predicted value (87.95%), indicating improved optical clarity. Figure 2 displays the response surface for emulsification time, illustrating the connection between formulation variables and the response.

**Table 5:** Single-sample t-test of the responses.

Response	Predicted value	Actual data	p-value
Transmittance	87.95	93.36 ± 0.25	0.021
pH	7.11	7.13 ± 0.01	0.236
Emulsification time	35.91	35.46 ± 0.08	0.072

A p-value < 0.05 was considered statistically significant.

Among the 17 experimental runs, formulations that met all acceptance criteria (transmittance ≥90%, pH 7.0–7.18, and emulsification time ≤60 seconds) were identified as samples 4, 5, 10, 12, 13, 16, and 17, making up 41.2% of the design space. These acceptable formulations had compositional ranges of 14.29–16.70% CLO, 65.23–70.82% Tween 80, and 14.29–20.00% PEG 400. The remaining 10 formulations (samples 1, 2, 3, 6, 7, 8, 9, 11, 14, and 15) failed to meet one or more specifications, mainly because their transmittance values were below 90%.

### Characteristics and stability evaluation of CLON

The optimized CLON formulation showed an average DS of 25.19 ± 2.31 nm, confirming the formation of a nanoscale delivery system with a high surface area. The polydispersity index (PDI) was 0.30 ± 0.04, indicating a fairly narrow and uniform DS distribution. The ZP was −45.07 ± 8.15 mV, implying strong electrostatic repulsion between droplets and supporting the stability of the nanoemulsion system.

**Figure 3:**
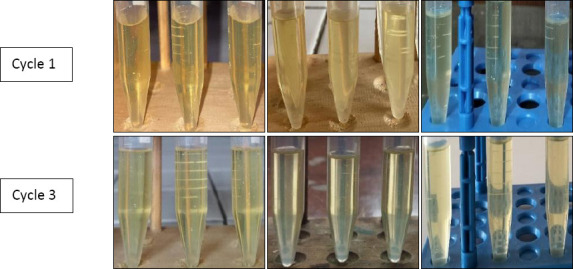
Stability of clove leaf oil nanoemulsion on heating–cooling (a), freeze–thaw (b), and centrifugation test.

**Figure 4:**
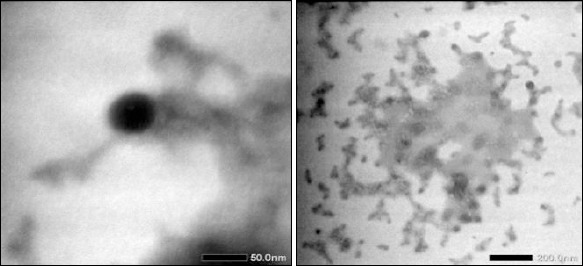
Transmission electron microscopy images of clove leaf oil nanoemulsion droplets with scale bars of 50 nm (left) and 200 nm (right).

Thermodynamic stability tests showed that the optimized formulation remained stable through all heating–cooling cycles, centrifugation, and freeze–thaw tests. No creaming, phase separation, or cracking was observed, indicating strong resistance to thermal and mechanical stress. These findings suggest that the formulation is suitable for storage in farm conditions where temperature fluctuations and handling are common (Figure 3). Morphological analysis using TEM revealed spherical nanoemulsion droplets with scale bars of 50 and 200 nm (Figure 4).

### Antimicrobial activity

The antimicrobial activity of CLON against bacteria associated with bovine mastitis showed promising results. CLON concentration had a highly significant impact (p < 0.01) on the inhibition zone diameter for all tested microorganisms (Table 6). Post hoc analysis with Duncan’s multiple range test revealed distinct groups marked by superscript letters.

**Table 6:** Inhibition zone of CLON against bovine mastitis-associated microorganisms.

Microorganisms	PV-I 1%	Exc. C	CLON 0%	CLON 20%	CLON 40%	CLON 60%	CLON 80%	CLON 100%
*S. aureus*	14.32 ± 0.11^b^	0.00 ± 0.00^f^	0.00 ± 0.00^f^	4.02 ± 0.01^e^	7.13 ± 0.01^d^	12.05 ± 0.70^c^	13.95 ± 0.20^b^	16.25 ± 0.05^a^
*E. coli*	15.20 ± 0.16^a^	0.00 ± 0.00^f^	0.00 ± 0.00^f^	4.42 ± 0.13^e^	6.45 ± 0.19^d^	10.12 ± 0.29^c^	13.10 ± 0.25^b^	15.53 ± 0.06^a^
*C. albicans*	14.93 ± 0.06^a^	0.00 ± 0.00^f^	0.00 ± 0.00^f^	5.40 ± 0.32^e^	7.63 ± 0.21^d^	9.80 ± 0.60^c^	12.09 ± 0.45^b^	14.45 ± 0.43^a^

All data are in mm. PV-I = Povidone iodine, Exc. C = Excipient control (Tween 80 + PEG 400), CLON = Clove leaf oil nanoemulsion. Different letters within the same row indicate significant differences (p < 0.05). *S. aureus* = *Staphylococcus aureus*, *E. coli* = *Escherichia coli*, *C. albicans* = *Candida albicans*.

Lower CLON concentrations resulted in smaller inhibition zones. Concentrations below 60% showed gradually smaller zones, ranging from about 4.02 to 7.63 mm. In contrast, higher concentrations (60%–100%) produced stronger inhibition zones ranging from approximately 9.80 to 16.25 mm. CLON at 100% consistently created the largest inhibition zones and was not significantly different from povidone iodine (PV-I) for *E. coli* and *C. albicans* (sharing superscript “a”). Additionally, CLON at 80% showed antimicrobial activity comparable to PV-I against *S. aureus* (both marked with superscript “b”). The excipient control and CLON at 0% showed no inhibitory activity (0.00 mm), confirming that the antimicrobial effect came from the clove leaf oil component. Based on these results, CLON at 100% was chosen for subsequent MIC and MBC tests.

The MIC and MBC values of CLON were 12.5% and 50% (v/v), respectively, for all tested microorganisms (Table 7). The MIC indicates the lowest concentration that produces ≥90% growth inhibition, while the MBC indicates the concentration that results in ≥99.9% microbial killing. The MBC/MIC ratio of 4:1 classifies CLON as bactericidal. In comparison, PV-I showed lower MIC (0.13–0.25%) and MBC (0.5%) values, corresponding to approximately 50–96-fold higher potency for MIC and 100-fold higher potency for MBC. The consistent MIC and MBC values across Gram-positive (*S. aureus*), Gram-negative (*E. coli*), and yeast (*C. albicans*) species indicate broad-spectrum antimicrobial activity. Based on these results, the MIC value (12.5%) was selected as the reference concentration for the killing-time assay, with treatments set at 0% (negative control), 1× MIC (12.5%), 2× MIC (25%), and 3× MIC (37.5%).

**Table 7:** MIC and MBC comparison of PV-I 1% and CLON.

Microorganisms	MIC (%) PV-I	MIC (%) CLON	MBC (%) PV-I	MBC (%) CLON
*S. aureus*	0.25	12.5	0.5	50
*E. coli*	0.25	12.5	0.5	50
*C. albicans*	0.13	12.5	0.5	50

*C. albicans* = *Candida albicans*. *E. coli* = *Escherichia coli*, CLON = Clove leaf oil nanoemulsion. MBC = Minimum bactericidal concentration, MIC = Minimum inhibitory concentration, PV-I = Povidone iodine, *S. aureus* = *Staphylococcus aureus*, No batch-to-batch variability was observed (SD = 0.00).

**Figure 5:**
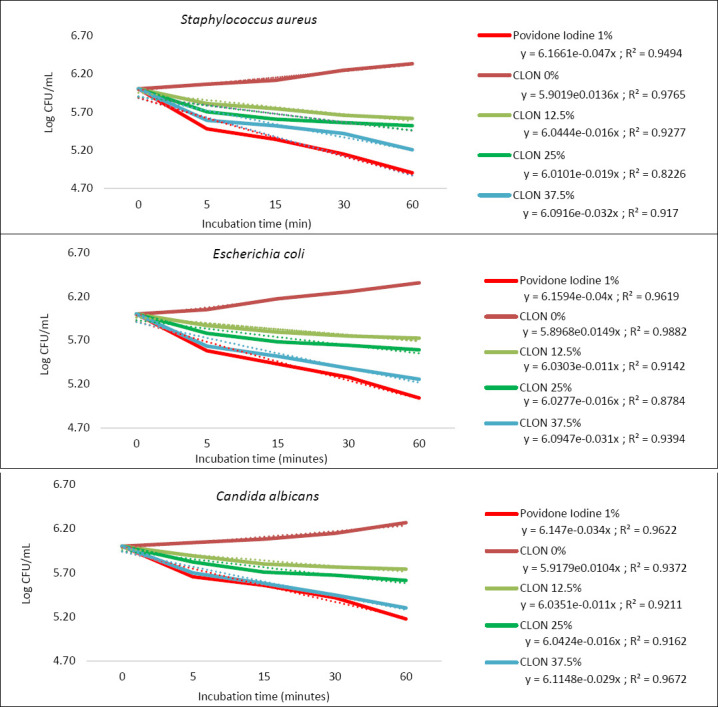
Killing-time assay of clove leaf oil nanoemulsion and 1% povidone iodine against *Staphylococcus aureus*, *Escherichia coli*, and *Candida albicans*.

The killing-time assay showed concentration- and time-dependent antimicrobial kinetics for all tested microorganisms (Figure 5, Table 8). The negative control (CLON 0%) exhibited increasing microbial counts over time, confirming active microbial growth without treatment. At 1× MIC (12.5% CLON), moderate antimicrobial activity was observed with log_10_ reductions of 3.73 ± 0.01, 2.97 ± 0.10, and 2.98 ± 0.10 log_10_ CFU/mL for *S. aureus*, *E. coli*, and *C. albicans*, respectively, after 60 min. At 2× MIC (25% CLON), enhanced killing was seen with reductions of 4.12 ± 0.17, 3.76 ± 0.20, and 3.73 ± 0.14 log_10_ CFU/mL, surpassing the ≥3 log_10_ CFU/mL reduction threshold required for disinfectant effectiveness. At 3× MIC (37.5% CLON), the highest antimicrobial activity was observed, with reductions of 5.20 ± 0.05, 5.14 ± 0.12, and 5.06 ± 0.10 log_10_ CFU/mL, nearing the efficacy of PV-I 1% (5.80 ± 0.08, 5.61 ± 0.14, and 5.34 ± 0.16 log_10_ reductions).

**Table 8:** Antimicrobial activity parameters of CLON.

Treatment	*S. aureus* Log Red	*S. aureus* AUKC	*E. coli* Log Red	*E. coli* AUKC	*C. albicans* Log Red	*C. albicans* AUKC
PV-I 1%	5.80 ± 0.08	123.58 ± 6.29	5.61 ± 0.14	139.50 ± 8.66	5.34 ± 0.16	157.61 ± 9.27
CLON 0%	NC	NC	NC	NC	NC	NC
CLON 12.5%	3.73 ± 0.01	233.12 ± 0.44	2.97 ± 0.10	262.55 ± 4.20	2.98 ± 0.10	262.76 ± 4.19
CLON 25%	4.12 ± 0.17	213.57 ± 7.74	3.76 ± 0.20	230.71 ± 8.82	3.73 ± 0.14	233.13 ± 5.89
CLON 37.5%	5.20 ± 0.05	162.48 ± 3.64	5.14 ± 0.12	166.16 ± 7.01	5.06 ± 0.10	172.70 ± 5.60

AUKC: Area under the killing curve (Log_10_ CFU min./mL), *C. albicans* = *Candida albicans*. *E. coli* = *Escherichia coli*, CLON = Clove leaf oil nanoemulsion, Log Red: Log_10_ reduction at 60 min (Log_10_ CFU/mL), NC = Not calculated because the curve showed an increasing trend, PV-I: Povidone iodine, *S. aureus* = *Staphylococcus aureus*. Values are Mean ± SD (n = 3).

*S. aureus* showed the fastest killing rate among the tested microorganisms, followed by *E. coli* and *C. albicans*, consistent with the susceptibility pattern observed in the inhibition zone assay. Analysis of the AUKC, which combines both the magnitude and rate of killing over the 60-min exposure, confirmed this concentration-dependent trend. PV-I 1% exhibited the lowest AUKC values (123.58–157.61 log_10_ CFU min./mL), followed by CLON 37.5% (162.48–172.70 log_10_ CFU min./mL), CLON 25% (213.57–233.13 log_10_ CFU min./mL), and CLON 12.5% (233.12–262.76 log_10_ CFU min./mL).

## DISCUSSION

### Formulation screening and selection of CLON composition

The initial screening of the oil and Smix (surfactant and co-surfactant) ratios in the CLON formulation (Table 2) showed that transmittance increased with higher Tween 80 concentrations. The 1:4:1 ratio produced transmittance values above 90% at the lowest Tween 80 level among the acceptable formulations and was therefore chosen for further optimization. Kaur *et al*. [19] similarly reported that an oil:Tween 80 ratio of 1:4 was optimal for nanoemulsion formation. Lechuga *et al*. [20] emphasized that lowering surfactant concentration is important for enhancing the safety profile of pharmaceutical formulations. Thus, the selected formulation achieved adequate emulsification while balancing safety and functional performance. Jia *et al*. [21] further indicated that optimizing oil and Smix ratios should aim to produce stable, environmentally adaptable, and cost-effective emulsions.

### Optimization of CLON using I-optimal mixture design

The I-optimal mixture design was chosen because it reduces the average prediction variance across the design space and is particularly well-suited for formulation optimization studies that require accurate prediction of response variables [15]. The optimization experiment produced formulations with transmittance values ranging from 55.34% to 93.54% (Table 3), showing that only specific compositional regions yield clear nanoemulsions. Statistical analysis verified that the developed linear model was valid and significant, with a non-significant lack-of-fit test (Table 4). Additionally, the close match between predicted R² and adjusted R² values (difference <0.2) confirmed that the model has adequate predictive capability without overfitting [22].

The model equations (Table 4) revealed that Tween 80 had the most significant impact on transmittance due to its role in lowering interfacial tension between oil and water phases. Conversely, CLO primarily influenced variations in emulsification time because higher oil concentrations require more surfactant coverage for stable emulsification. These results show that the differences among formulations reflect the system’s natural sensitivity to compositional changes rather than a flaw in the formulation strategy. Such predictive robustness is crucial for guiding scale-up and industrial production [23].

From a practical standpoint, formulations with CLO levels above approximately 18% consistently failed the transmittance requirement because the available surfactant was not enough to fully stabilize the oil phase. Similarly, reducing Tween 80 concentrations below about 63% led to incomplete emulsification and longer processing times. These results highlight Tween 80 as the most critical component that needs precise control during scale-up manufacturing.

### pH compatibility and emulsification behavior

The pH range of the optimized formulations (7.02–7.16) is especially relevant for mastitis teat disinfection because it closely matches the physiological pH of bovine teat skin (7.00–7.18), as reported by Fox *et al*. [25]. Pour *et al*. [24] observed that topical disinfectant formulations should stay compatible with skin pH to reduce irritation. In contrast, conventional iodophor-based teat disinfectants usually have acidic pH levels (3–6), which may cause skin irritation during long-term use [26]. Therefore, the near-neutral pH of CLON offers a significant advantage in preserving skin integrity during repeated applications.

The emulsification time ranged from 35.4 to 37.4 s, indicating rapid and efficient nanoemulsion formation. According to Taha *et al*. [27], longer emulsification times are generally associated with reduced emulsion stability because they reflect difficulty in achieving uniform phase dispersion. The short emulsification time observed in this study, therefore, indicates favorable emulsification kinetics and formulation stability.

Diagnostic plots further verified the reliability of the statistical models (Figure 1). The standardized residuals exhibited a linear distribution pattern, indicating normality, while residual-versus-run plots displayed a random distribution, confirming independence and homoscedasticity of the residuals [28]. These statistical diagnostics support the robustness of the developed formulation models.

### Physicochemical characteristics and stability of CLON

The optimized CLON formulation showed a mean DS of 25.19 ± 2.31 nm, confirming the successful formation of a nanoemulsion system. The polydispersity index (0.30) indicated a relatively narrow DS distribution, while the strongly negative ZP (−45.07 ± 8.15 mV) suggested robust electrostatic repulsion between droplets. These features are consistent with previously reported clove oil nanoemulsions, such as those described by Shahavi *et al*. [29].

Ultrasonication likely played a significant role in reducing DS. While mechanical stirring alone generally produces droplets in the 100–500 nm range, ultrasonication can markedly decrease DS through acoustic cavitation effects. Previous studies have reported droplet sizes between 68 and 189 nm with ZP values from −44 to −55 mV when sonication is used [30, 31].

Although Tween 80 is a non-ionic surfactant, the strongly negative ZP observed in CLON may result from hydroxyl ion adsorption at the oil–water interface or from anionic impurities like free fatty acids in the oil phase, rather than surfactant ionization itself [32, 33]. PEG 400 further increased system stability by providing steric stabilization, which improved droplet uniformity and prevented coalescence [5, 34].

Thermodynamic stability testing showed that CLON remained stable under freezing (−4°C), heating (45°C), and centrifugation stress conditions. No creaming, cracking, or phase separation was observed, indicating strong physical stability. This stability is especially important for farm-level applications where products might face varying storage and handling conditions.

TEM images showed spherical droplets with a clear shape (Figure 4). Small droplet clusters seen in the micrographs probably resulted from dehydration artifacts during sample preparation, not from instability in the emulsion system. This is supported by the strong ZP and excellent thermodynamic stability observed during characterization [35–37].

### Antimicrobial activity against mastitis-associated pathogens

CLON concentrations from 60% to 100% caused moderate to strong inhibition of the tested microorganisms. According to Humphries *et al*. [38], inhibition zones larger than 14 mm indicate sensitivity, while zones between 9 and 14 mm show intermediate susceptibility. The lack of antimicrobial activity in the excipient control confirmed that the inhibitory effect was due to CLO, not the surfactant system.

Differences in antimicrobial susceptibility among the tested microorganisms can be explained by structural variations in their cell envelopes. Gram-positive bacteria, such as *Staphylococcus aureus*, have thick peptidoglycan layers but lack an outer membrane, making them more vulnerable to lipophilic antimicrobial compounds like eugenol. In contrast, Gram-negative bacteria, such as *Escherichia coli*, have an additional outer membrane containing lipopolysaccharides that serves as a permeability barrier against hydrophobic antimicrobial agents [39, 40]. The yeast *Candida albicans* has a complex cell wall made up of chitin, β-glucan, and mannoproteins, which can further restrict antimicrobial penetration [41].

The nanoscale DS probably enhanced antimicrobial activity by increasing surface area, improving water dispersibility of hydrophobic compounds, and facilitating interaction with microbial membranes [41, 42]. Previous studies have shown that nanoemulsification can lower MIC values of essential oils by about 50% compared to non-nanoemulsified oils [43, 44].

### Killing kinetics and disinfectant performance

The killing-time assay showed clear concentration-dependent antimicrobial kinetics. A 25% CLON concentration (2× MIC) achieved over a 3-log_10_ CFU/mL reduction within 60 minutes for all tested microorganisms, meeting the efficacy threshold commonly used for disinfectant evaluation [45]. Faster killing of *S. aureus* compared to *E. coli* aligned with structural susceptibility differences between Gram-positive and Gram-negative bacteria. The rapid bactericidal activity observed aligns with how eugenol works, which damages cell membranes and causes quick leakage of internal contents instead of slow metabolic inhibition [41, 42, 46]. AUKC analysis also confirmed the antimicrobial effect depends on concentration, with CLON 37.5% nearly matching the activity of 1% povidone iodine. Considering both antimicrobial efficacy and practical formulation considerations, the 25% CLON concentration offers a promising balance between antimicrobial performance and resource efficiency. This finding is especially relevant for teat disinfection practices, where short contact times are commonly used during pre-milking sanitation.

### Mechanism of antimicrobial action and phytochemical synergy

**Figure 6:**
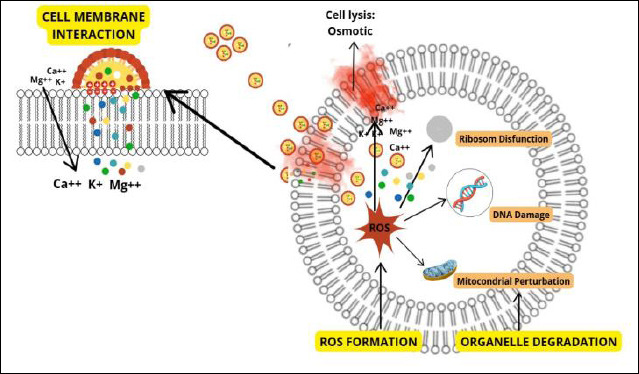
Overview of the multiple simultaneous antimicrobial mechanisms of clove leaf oil nanoemulsion.

The antimicrobial activity of CLON results from its complex phytochemical composition. GC–MS analysis showed that eugenol (72.90%) was the main compound, followed by β-caryophyllene (12.00%), eugenyl acetate (3.84%), caryophyllene oxide (2.12%), α-copaene (1.88%), and various minor terpenoids (Table 1). Eugenol mainly works by disrupting membranes, inhibiting ATPase, producing reactive oxygen species, and inactivating enzymes (Figure 6).

Additional phytochemicals may work together synergistically. β-Caryophyllene may improve membrane permeability and influence inflammatory responses, while eugenyl acetate and caryophyllene oxide may provide additional antimicrobial actions [46, 47]. This multi-compound makeup offers a potential advantage of plant-derived antimicrobials, as targeting multiple cellular pathways simultaneously decreases the chances of resistance developing [3].

### Implications for mastitis control and future research

Overall, the optimized CLON formulation showed favorable physicochemical properties, strong thermodynamic stability, and broad-spectrum *in vitro* antimicrobial activity against major microorganisms associated with bovine mastitis. These findings suggest that CLON is a promising plant-derived alternative for teat disinfection.

However, further research is needed before practical application can be considered. Clinical mastitis isolates may exhibit different susceptibility patterns compared to reference strains, and in vivo studies are essential to determine whether the *in vitro* activity observed translates into effective mastitis prevention. Future work should also compare the effects with non-nanoemulsified CLO to measure the specific enhancement from nanoemulsification, evaluate shorter contact times relevant to field conditions, and assess the economic feasibility for large-scale dairy farm use.

## CONCLUSION

This study successfully developed and optimized CLON using SNEDDS combined with an I-optimal mixture design. The optimized formulation contained 14.29% CLO, 68.56% Tween 80, and 17.15% PEG 400, resulting in a stable nanoemulsion with desirable physicochemical properties, including high transmittance (93.36 ± 0.25%), physiological pH (7.13 ± 0.01), rapid emulsification time (35.46 ± 0.08 s), nanoscale particle size (25.19 ± 2.31 nm), narrow size distribution (PDI 0.30 ± 0.04), and strong electrostatic stability (ZP −45.07 ± 8.15 mV). The formulation remained stable during centrifugation, heating–cooling cycles, and freeze–thaw conditions, confirming excellent thermodynamic stability. Antimicrobial tests showed broad-spectrum *in vitro* activity against mastitis causing pathogens (*S. aureus*, *E. coli*, and *C. albicans*). CLON had MIC and MBC values of 12.5% and 50% (v/v), respectively, indicating bactericidal activity (MBC/MIC ratio 4:1). In the killing-time assay, 25% CLON achieved more than a 3 log_10_ CFU/mL reduction within 60 min for all tested microorganisms, demonstrating effective disinfectant-level antimicrobial activity.

From a practical standpoint, the formulation offers several advantages for mastitis control. The near-neutral pH matches the physiological pH of bovine teat skin, which may help reduce irritation caused by repeated use of acidic iodophor disinfectants like PV-I. The nanoscale DS improves the dispersibility and antimicrobial effectiveness of CLO, while the demonstrated thermodynamic stability indicates that the formulation can remain stable under the variable storage and handling conditions common on dairy farms. Additionally, using CLO derived from fallen leaves provides a sustainable and cost-effective raw material compared to clove bud oil.

A key strength of this study is its systematic formulation approach that combines SNEDDS technology with statistical optimization, allowing for precise control of nanoemulsion features and the identification of a reliable formulation design space. Moreover, the antimicrobial assessment used additional methods such as inhibition zone analysis, MIC/MBC tests, and killing-time kinetics, offering thorough evidence of CLON’s antimicrobial potential.

However, several limitations should be recognized. The antimicrobial assessment was conducted solely under *in vitro* conditions using reference strains, which might not fully reflect the diversity and resistance profiles of field mastitis isolates. Additionally, this study did not include a direct comparison with non-nanoemulsified CLO, which restricts the ability to determine the specific improvement due to nanoemulsification. Long-term shelf-life stability, compatibility with routine dairy farm disinfection protocols, and economic feasibility for large-scale use were also not assessed.

Future research should therefore focus on verifying CLON’s effectiveness against clinical mastitis isolates, testing shorter contact times relevant to field disinfection practices, and conducting in vivo studies to assess its ability to reduce intramammary infections and somatic cell counts. Additional studies should also examine long-term storage stability, optimize formulation delivery as teat dip or spray products, and perform cost–benefit analyses for commercial use.

In conclusion, the optimized CLON formulation showed good physicochemical stability and promising *in vitro* antimicrobial activity against microorganisms associated with mastitis. These findings suggest that CLON is a promising plant-derived candidate for teat disinfection. However, further translational and field studies are needed to verify its safety, effectiveness, and practical use in commercial dairy production conditions.

## DATA AVAILABILITY

Supplementary data can be made available from the corresponding author upon request.

## AUTHORS’ CONTRIBUTIONS

TES: Conceptualization, methodology, supervision, resources, project administration, writing – original draft, review, and editing. ANS: Investigation, data acquisition, formal analysis, data interpretation, visualization, writing – original draft, project administration, funding acquisition. SS and LER: Supervision, validation, methodology, writing – review and editing. All authors contributed to the study design, critically reviewed the manuscript, and approved the final version of the manuscript.
